# Circulating cell death products predict clinical outcome of colorectal cancer patients

**DOI:** 10.1186/1471-2407-9-88

**Published:** 2009-03-23

**Authors:** Pim J Koelink, Cornelis BHW Lamers, Daan W Hommes, Hein W Verspaget

**Affiliations:** 1 Department of Gastroenterology and Hepatology, Leiden University Medical Centre, C4-P, P.O. Box 9600, 2300 RC Leiden, The Netherlands

## Abstract

**Background:**

Tumor cell death generates products that can be measured in the circulation of cancer patients. CK18-Asp396 (M30 antigen) is a caspase-degraded product of cytokeratin 18 (CK18), produced by apoptotic epithelial cells, and is elevated in breast and lung cancer patients.

**Methods:**

We determined the CK18-Asp396 and total CK18 levels in plasma of 49 colorectal cancer patients, before and after surgical resection of the tumor, by ELISA. Correlations with patient and tumor characteristics were determined by Kruskal-Wallis H and Mann-Whitney U tests. Disease-free survival was determined using Kaplan-Meier methodology with Log Rank tests, and univariate and multivariate Cox proportional hazard analysis.

**Results:**

Plasma CK18-Asp396 and total CK18 levels in colorectal cancer patients were related to disease stage and tumor diameter, and were predictive of disease-free survival, independent of disease-stage, with hazard ratios (HR) of patients with high levels (> median) compared to those with low levels (≤ median) of 3.58 (95% CI: 1.17–11.02) and 3.58 (95% CI: 0.97–7.71), respectively. The CK18-Asp396/CK18 ratio, which decreased with tumor progression, was also predictive of disease-free survival, with a low ratio (≤ median) associated with worse disease-free survival: HR 2.78 (95% CI: 1.06–7.19). Remarkably, the plasma CK18-Asp396 and total CK18 levels after surgical removal of the tumor were also predictive of disease-free survival, with patients with high levels having a HR of 3.78 (95% CI: 0.77–18.50) and 4.12 (95% CI: 0.84–20.34), respectively, indicating that these parameters can be used also to monitor patients after surgery.

**Conclusion:**

CK18-Asp396 and total CK18 levels in the circulation of colorectal cancer patients are predictive of tumor progression and prognosis and might be helpful for treatment selection and monitoring of these patients.

## Background

Death of tumor cells generates detectable protein products in the patient's circulation, which may be used for cancer diagnostics and/or monitoring of therapy efficacy[1]. Apoptosis is a form of regulated cell death that is characterized by specific structural changes, mediated by proteases of the caspase family [2]. Caspase activity itself or the presence of specific degradation products can be used for the detection of tumor cell apoptosis.

The M30 antibody detects a caspase-degraded product, CK18-Asp396, of the important cytoskeletal protein cytokeratin 18 (CK18) of epithelial cells, which is expressed by most carcinomas, including those of breast, prostate, lung and colon [3]. Immunohistochemistry with the M30 antibody has been shown to be as specific as the morphological detection or TdT-mediated dUTP-biotin nick end-labelling (TUNEL) technique to establish of apoptosis in tissue [3-5]. Importantly, the levels of CK18-Asp396 can also be determined in the circulation by a specific ELISA, allowing the detection of tumor cell apoptosis in the serum/plasma of cancer patients [6,7]. However, CK18-Asp396 detection in the plasma is not tumor specific, healthy controls have background levels, due to apoptosis of normal epithelial cells. Circulating CK18-Asp396 levels were found to be elevated in patients with lung and breast cancer, and were predictive to survival or recurrence outcome [8,9]. In addition, circulating CK18-Asp396 levels increased shortly after chemotherapy in hormone-refractory prostate cancer and lung cancer, implying that this was a result of chemotherapy-induced tumor cell apoptosis [9-11].

During non-programmed cell death, i.e., necrosis, intact CK18 of epithelial tumor cells is released into the circulation, which can be measured by a total CK18 ELISA, that also detects the CK18-Asp396 [11]. Cytokeratins and their cleaved forms are secreted in aggregates into the circulation and both CK18-Asp396 and total CK18 levels in human plasma samples have a long-term stability when stored at -80°C [12].

The ratio between CK18-Asp396 and total CK18 levels in the circulation depends on the balance between caspase-mediated apoptosis and non-proteolytic necrosis. This balance might be an important factor and denominator to select patient for treatment that induces necrosis vs treatment that increases apoptosis. For instance, docetaxel treatment increased levels of CK18-Asp396 in the serum of breast cancer patients, indicating apoptotic death of tumor cells, while cyclophosphamide/epirubicin/5-fluorouracil treatment increased total CK18 levels, indicating necrotic death of tumor cells [13]. The increase of total CK18 serum levels correlated to the clinical therapy response [13]. The CK18-Asp396/CK18 ratio was shown to be decreased, i.e., more necrosis over apoptosis, in endometrial cancer stage III/IV when compared with stage II, indicating less apoptosis and/or more necrosis during tumor progression [11]. In the present study, we determined the CK18-Asp396 and total CK18 levels in plasma of 49 colorectal cancer patients and found these levels to predict clinical outcome of these patients.

## Methods

### Patients, plasma and tissue collection

The study population consisted of 49 colorectal cancer patients that did not receive pre-operative treatment and had been admitted to the Leiden University Medical Centre for surgical resection. Citrate plasma samples were collected, with informed consent of the patients, before resection (P1, pre-operative), shortly after surgical resection (P2) and about 4.5 months after the operation (P3, post-operative), when feasible. Citrate plasma samples were collected before 9.00 a.m. under fasting conditions and stored at -70°C. Fresh tissue was collected from the surgical specimens immediately after resection, and attention was paid to collect material from the non-necrotic part of the tumor. Normal mucosa samples were obtained at a distance of approximately 10 cm from the tumor. Tissue samples were also frozen and stored at -70°C until use. Macroscopic (diameter and localization of the tumor) as well as microscopic data were assessed, including classification according to the WHO. Colonic cancers were classified as being proximal or distal to, and including, the splenic flexure. Follow-up information, including post-operative adjuvant therapy, was available for a period up to 8 years. The study was performed according to the guidelines of the Medical Ethics Committee of the Leiden University Medical Centre in compliance with the Helsinki Declaration.

### Tissue homogenization and protein determination

Frozen tissue specimens were weighed and homogenized on ice for 2 minutes in 1 ml Tris-HCl, 0.1% Tween 80, pH 7.5 per 60 mg tissue using a Potter device (B Braun, Germany), and centrifuged twice at 8000 × *g *for 2.5 min at 4°C. Protein content was measured according to Lowry *et al. *and standardized by bovine serum albumin [14].

### CK18-Asp396 and total CK18 detection

For the detection of CK18-Asp396 and total CK18 in the plasma of colorectal cancer patients commercially available immunosorbent sandwich ELISAs were used, according to manufacturer's instructions (M30-apoptosense ELISA and M65 ELISA, Peviva, Sweden). For the determination of CK18-Asp396 and total CK18 levels in tissues 1 μg/μl protein homogenates were diluted up to 1000 times, dependent on antigen levels. The antigen levels in the plasma were expressed as U/L and antigen levels in tumor tissue or normal mucosa were expressed as U/mg protein.

### Statistical analysis

Statistical analysis was performed with Statistical Package for Social Sciences (SPSS) statistical software (version 12.0 for Windows, SPSS Inc, Chicago, IL). For relations between CK18-Asp396 or total CK18 antigen levels in P1, P2 and P3 Spearman correlation and Wilcoxon signed-rank tests were used. For the relation with patient characteristics non-parametric Spearman correlation, Kruskal-Wallis H and Mann-Whitney U tests were used, because the study parameters did not follow a normal distribution. Disease-free survival was estimated using Kaplan-Meier methodology with cancer-related death and local or distant recurrence as events, dichotomized for CK18-Asp396, total CK18 or CK18-Asp396/CK18 ratio levels, and Log Rank tests. Univariate and multivariate Cox proportional hazard models were used to explore the association of markers with disease-free survival. Kaplan Meier graphs were made with Graphpad Prism (version 4.0, Graphpad Prism Inc., La Jolla, CA, USA) software.

## Results

### CK18-Asp396 and total CK18 levels in plasma of colorectal cancer patients

Overall the plasma CK18-Asp396 and total CK18 levels correlated very well with each other, in plasma before, as well as shortly and longer after tumor resection (overall Spearman correlation coefficient Rho = 0.64, p < 0.0001).

The CK18-Asp396 and total CK18 plasma values increased shortly after surgical resection of the tumor and dropped to about pre-operative values longer after surgery (Table 1).

**Table 1:** CK18-Asp396 and total CK18 levels in plasma of CRC patients.

	P1 (n = 49)	P2 (n = 20)	P3 (n = 28)
Mean time to operation (days, range)	-13 (-50-0)	+20 (7–60)	+137 (82–364)

*CK18-Asp396 level (U/l)*			
Median (IQR)	59.1 (41.5–88.2)	74.8 (39.7–107.9)	55.6 (42.1–79.9)
P-value		**0.02 vs P1**	0.11 vs P2

*Total CK18 level (U/l)*			
Median (IQR)	260.5 (181.6–378.3)	308.1 (208.7–492.3)	257.2 (183.8–457.2)
**P-value**		0.08 vs P1	**0.05 vs P2**

CK18-Asp396 and total CK18 levels in plasma of colorectal cancer patients. Wilxocon signed-rank tests were used to compare paired observations. P ≤ 0.05 were considered significant, shown in bold. P1 = pre-operative plasma, P2 = post-operative plasma shortly after operation, and P3 = post-operative plasma longer after operation.

### Correlation pre-operative plasma CK18-Asp396 and CK18 levels with clinico-pathological patient parameters

**Figure 1:**
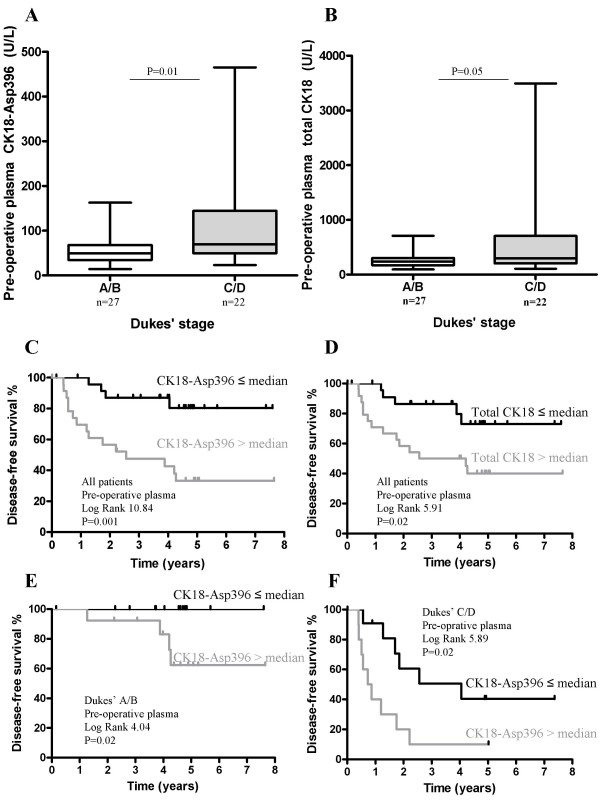
**Pre-operative plasma CK18-Asp396 and total CK18 levels, and survival**. CK18-Asp396 and total CK18 levels in pre-operative plasma of all colorectal cancer patients with a Dukes' A/B vs Dukes' C/D stage carcinoma (P1, **A **and **B**). Box plots with line indicating median value, box indicating IQR and bars indicating the range. Kaplan Meier disease-free survival curves of all colorectal cancer patients, groups divided upon median values of CK18-Asp396 (**C**) and total CK18 levels (**D**) in pre-operative plasma (P1). Patients were also subdivided in Dukes' A/B (**E**, n = 22) and Dukes' C/D (**F**, n = 27) stage carcinoma.

The clinico-pathological parameters of the 49 patients are shown in Table 2. CK18-Asp396 and total CK18 plasma levels did not correlate with localization of the tumor, also not within the patients with only colonic tumors (not shown). Male and female colorectal cancer patients had similar plasma CK18-Asp396 and total CK18 levels, and these were not correlated with patients' age. The CK18-Asp396 and total CK18 plasma values were higher in patients with more advanced tumor stages (Figure 1A and 1B, p = 0.01 and p = 0.05, respectively). CK18-Asp396 levels correlated with the diameter of the tumor (Spearman correlation coefficient Rho = 0.35, p = 0.02). Both CK18-Asp396 and total CK18 levels were significantly higher in the eight patients with a Dukes' D tumor in which the tumor was not (or not curatively) resected.

**Table 2:** Clinico-pathological characteristics and pre-operative plasma levels

Patient and tumor characteristics	No of patients (%) Total n = 49	Plasma CK18-Asp396 (U/l) Median (IQR)	*P-value*	Total CK18 plasma level (U/l) Median (IQR)	*P-value*
Gender			0.87		0.92
Male	31 (63)	61.1 (41.8–88.2)		260.5 (174.1–382.8)	
Female	18 (37)	50.5 (37.6–110.2)		252.9 (184.5–352.1)	
Age (median 68 year, range 31–84)			0.32		0.38
≤ median	25 (51)	59.1 (15.1–99.0)		288.6 (181.6–403.5)	
> median	24 (49)	55.6 (34.2–74.0)		232.4 (177.3–328.6)	
Location			0.63		0.25
Colon	38 (78)	59.4 (40.5–102.8)		268.6 (199.7–391.1)	
Rectum	11 (22)	56.4 (43.3–68.4)		208.4 (174.1–377.9)	
Dukes' stage			**0.01**		**0.05**
A/B	27 (55)	49.3 (34.2–67.8)		235.0 (170.5–303.5)	
C/D	22 (45)	69.2 (49.7–114.5)		295.8 (202.3–706.3)	
WHO classification			0.43		0.56
Adenocarcinoom	45 (92)	59.1 (39.9–82.2)		265.4 (186.7–378.3)	
Mucinous carcinoom	4 (8)	77.8 (48.9–177.7)		211.2 (172.0–363.1)	
Tumor diameter (median 4.5 cm, range 2–13.5)*			**0.03**		0.07
≤ median	24 (55)	49.1 (31.9–67.7)		232.4 (174.5–187.7)	
> median	20 (45)	64.7 (43.7–133.3)		287.3 (180.0–657.6)	
Surgery			**0.001**		**<0.001**
Yes, curative resection	41 (84)	51.1 (36.5–73.7)		235.0 (172.3–331.9)	
No, or palliative	8 (16)	162.9 (68.8–346.4)		971.7 (306.1–2290.4)	

Clinico-pathological characteristics and pre-operative CK18-Asp396 and total CK18 plasma levels. P-values were calculated with Kruskal-Wallis and Mann-Whitney tests and P ≤ 0.05 were considered significant, shown in bold. * Some values missing.

### Pre-operative plasma CK18-Asp396 and CK18 levels and disease-free survival

The disease-free survival of the patients with low CK18-Asp396 plasma levels before resection (≤ median) was significantly better compared with patients with high CK18-Asp396 plasma levels (Figure 1C). This was also the case for total CK18 plasma levels (Figure 1D). When the patients were subdivided in "early" and "advanced" tumors, Dukes' A/B stage carcinoma (n = 27) and C/D stage carcinoma (n = 22), the plasma CK18-Asp396 level was also prognostic within these subgroups (Figure 1E and 1F). Calculation of hazard ratios as estimates of relative risk of death or disease recurrence is shown in Table 3. Tumor progression, CK18-Asp396 and total CK18 levels in plasma before surgery were predictive of recurrence or death.

**Table 3:** Univariate and multivariate Cox regression analysis

Patient and tumor characteristics	*Univariate Hazard Ratio (95% confidence interval)*	*P-value*	*Multivariate Hazard Ratio (95% confidence interval)*	*P-value*
Gender		0.27		
Female	1 (ref)			
Male	1.79 (0.64–497)			
Age (median 68 year)		0.83		
≤ median	1 (ref)			
> median	1.10 (0.45–2.73)			
Location		0.32		
Colon	1 (ref)			
Rectum	0.54 (0.16–1.85)			
Dukes' stage		**<0.001**		**<0.001–0.021**
A/B	1 (ref)		1(ref)	
C/D	8.21 (2.70–24.97)		6.01–9.60 (1.32–33.31)	
Tumor diameter (median 4.5 cm)		0.32		
≤ median	1 (ref)			
> median	1.68 (0.61–4.65)			
WHO classification		0.82		
Adenocarcinoom	1 (ref)			
Mucinous carcinoom	0.79 (0.11–5.90)			
CK18-Asp396 plasma level P1		**0.003**		**0.03**
≤ median	1 (ref)		1 (ref)	
> median	5.28 (1.75–15.92)		3.58 (1.17–11.02)	
Total CK18 plasma level P1		**0.02**		0.055
≤ median	1 (ref)		1 (ref)	
> median	3.30 (1.19–9.16)		3.58 (0.97–7.71)	
CK18-Asp396/CK18 ratio P1		0.12		**0.04**
≤ median	2.09 (0.82–5.31)		2.78 (1.06–7.19)	
> median	1 (ref)		1 (ref)	
CK18-Asp396 plasma level P3		0.054		0.10
≤ median	1 (ref)		1 (ref)	
> median	4.71 (0.97–22.85)		3.78 (0.77–18.50)	
Total CK18 plasma level P3		0.055		0.08
≤ median	1 (ref)		1 (ref)	
> median	4.69 (0.97–22.72)		4.12 (0.84–20.34)	
CK18-Asp396/CK18 ratio P3		**0.04**		0.10
≤ median	5.28 (1.08–25.72)		3.96 (0.78–20.08)	
> median	1 (ref)		1 (ref)	

Cox hazards analysis of patient and tumor characteristics in relation to disease-free survival. Multivariate Cox hazards analysis of pre-operative or post-operative CK18-Asp396, total CK18 antigen level or CK18-Asp396/CK18 ratios combined with Dukes' stage. P ≤ 0.05 were considered significant, shown in bold.

**Figure 2:**
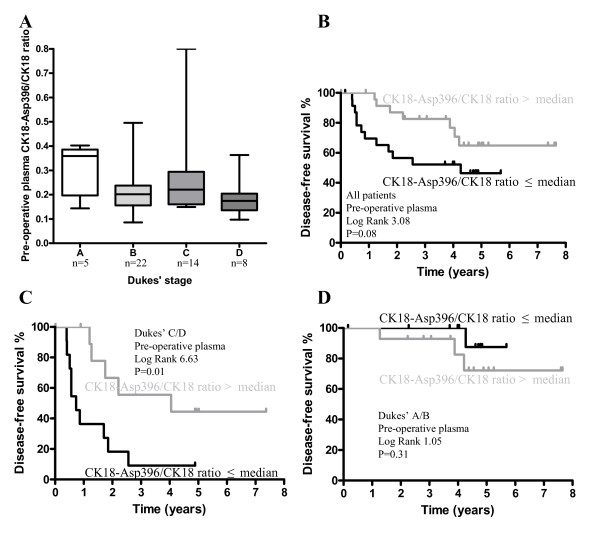
**Pre-operative plasma CK18-Asp396/CK18 ratios and survival**. Pre-operative plasma CK18-Asp396/CK18 ratios decrease with increasing Dukes' stage (P1, **A**). Box plots with line indicating median value, box indicating IQR and bars indicating the range. Kaplan Meier disease-free survival curves of all colorectal cancer patients, groups divided upon median pre-operative plasma CK18-Asp396/CK18 ratios (**B**). Patients were also subdivided in Dukes' C/D (**C**, n = 22) and Dukes' A/B stage carcinoma (**D**, n = 27).

The CK18-Asp396/CK18 ratio indicates the balance between caspase-mediated apoptosis and non-proteolytic necrosis. CK18-Asp396/CK18 ratios of pre-operative plasmas of colorectal cancer patients were calculated and a large variety was observed in different patients with a median ratio of 0.20 (IQR: 0.15–0.26). The CK18-Asp396/CK18 ratio tended to decrease with increasing Dukes' stage (Figure 2A), indicating more necrosis over apoptosis, during tumor progression. The disease-free survival of patients with high ratios tended to be better when compared with those with lower ratios (Figure 2B). This relation was particularly present in patients with Dukes' C/D stage carcinomas (Figure 2C), in contrast to patients with Dukes' A/B stage carcinomas where no such relation was found (Figure 2D).

### CK18-Asp396, CK18 levels and CK18-Asp396/CK18 ratios in post-operative plasma

**Figure 3:**
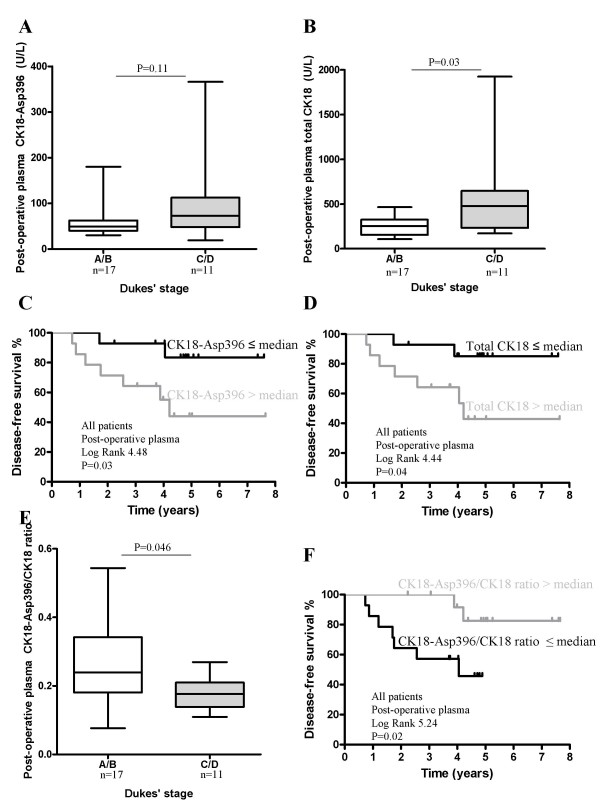
**Post-operative CK18-Asp396, total CK18 and CK18-Asp396/CK18 ratios levels, and survival**. Post-operative (P3) CK18-Asp396 and total CK18 levels of colorectal cancer patients with a Dukes' A/B vs Dukes' C/D stage carcinoma (**A **and **B**). Box plots with line indicating median value, box indicating IQR and bars indicating the range. Kaplan Meier disease-free survival curves of all colorectal cancer patients, groups divided upon median post-operative CK18-Asp396 (**C**, n = 28) and total CK18 levels (**D**, n = 28). Plasma CK18-Asp396/CK18 ratios were decreased in patients Dukes' C/D vs Dukes' A/B (**E**). Kaplan Meier disease-free survival curves of colorectal cancer patients, groups divided upon median post-operative plasma CK18-Asp396/CK18 ratios (**F**, n = 28).

Post-operative plasma CK18-Asp396 levels of patients about 4.5 months after resection of Dukes' C/D stage carcinomas were also somewhat, but not significantly, higher compared with patients with Dukes' A/B stage carcinomas (Figure 3A). The total CK18 plasma levels, however, were significantly higher in Dukes' C/D tumor patients compared with Dukes' A/B tumor patients (Figure 3B). High CK18-Asp396 levels as well as total CK18 plasma levels after tumor resection were associated with worse disease-free survival (Figure 3C and 3D), also found in the univariate Cox hazard analysis (Table 3).

CK18-Asp396/CK18 ratios in post-operative patients' plasma were in a similar range as in the patients' plasma before surgery, and also tended to decrease with increasing Dukes' stage, i.e., significantly higher in Dukes' A/B compared with Dukes' C/D patients (Figure 3E). The disease-free survival was again better in patients with high post-operative plasma CK18-Asp396/CK18 ratios (Figure 3F).

In 8 of the 28 patients post-operative treatment with radiotherapy and in only one patient treatment with combined radio-chemotherapy was started before collection of the 4.5 months plasma samples. There were no significant differences in post-operative plasma CK18-Asp396 and total CK18 levels, and in CK18-Asp396/CK18 ratios in patients receiving post-operative adjuvant therapy compared to the patients without adjuvant therapy (data not shown, Additional file 1).

### Multivariate Cox regression analysis

A multivariate Cox proportional hazards model of disease-free survival was used to evaluate whether the plasma CK18-Asp396 and total CK18 levels showed independent prognostic significance from tumor staging. These analyses showed that patients with high pre-operative CK18-Asp396 plasma levels have a 3.6 times increased relative risk of colorectal cancer-related-death or disease recurrence (Table 3), independent of Dukes' staging. For total CK18 levels, this was quite similar although just not significant. Patients with low pre-operative plasma CK18-Asp396/CK18 ratios had a 2.8 times increased risk to develop recurrence or death. Post-operative CK18-Asp396, total CK18 and CK18-Asp396/CK18 ratio levels all had similar prognostic significance, although not significant (Table 3).

### Correlation tumor and plasma CK18-Asp396 level

Corresponding colorectal tumor tissue was obtained in 40 cases. CK18-Asp396 levels were significantly (p = 0.05) higher in tumor tissue when compared with normal adjacent tissue with a median value of 2.1 (IQR: 0.3–7.7, n = 40) vs 1.8 (IQR: 0.1–4.2, n = 36) U/mg, respectively.

Tumor CK18-Asp396, total CK18 levels and CK18-Asp396/CK18 ratios were only found to correlate with tumor location, with rectal tumors having higher levels (Table 4). Surprisingly, CK18-Asp396 plasma levels showed a tendency to inversely correlate with the CK18-Asp396 level of the tumor (Rho = -0.307, p = 0.054). Tumor CK18-Asp396, total CK18 levels and CK18-Asp396/CK18 ratios were found to be not prognostic for disease-free survival (data not shown, Additional File 2).

**Table 4:** Clinico-pathological characteristics and tumor levels

*Patient and tumor characteristics*	*No of patients (%) Total n = 40*	*CK18-Asp396 level (U/mg) Median (IQR)*	*P-value*	*Total CK18 level (U/mg*) Median (IQR)	*P-value*	CK18-Asp396/*CK18 × 100 Median (IQR)*	*P-value*
Gender			0.94		0.67		0.96
Male	26	2.1 (0.2–7.7)		203.0 (64.0–318.2)		1.9 (0.3–3.7)	
Female	14	1.3 (0.2–10.3)		159.0 (33.2–273.5)		1.7 (0.2–3.6)	
Age (median 68 year)			0.98		0.29		0.77
≤ median	20	1.4 (0.3–6.1)		165.0 (53.0–331.7)		1.8 (0.5–3.4)	
> median	20	2.2 (0.2–9.7)		219.1 (57.0–248.9)		2.2 (0.2–3.8)	
Location			**0.003**		**0.03**		**0.005**
Colon	32	0.8 (0.2–5.7)		147.0 (47.1–243.3)		1.4 (0.2–2.4)	
Rectum	8	12.7 (3.5–19.5)		291.5 (214.6–369.7)		3.7 (2.8–6.3)	
Dukes' stage			0.79		0.49		0.90
A/B	25	1.6 (0.3–9.7)		177.1 (71.8–310.2)		1.8 (0.2–3.4)	
C/D	15	1.3 (0.1–5.9)		222.4 (32.7–252.1)		2.0 (0.4–3.8)	
WHO classification			0.90		0.49		0.94
Adenocarcinoom	37	1.6 (0.3–7.7)		190.3 (56.9–281.9)		1.9 (0.3–3.4)	
Mucinous carcinoom	3	1.1		65.4		1.7	
Tumor diameter (median 4.5 cm)*			0.37		0.43		0.31
≤ median	20	4.3 (0.3–10.3)		203.0 (56.9–310.2)		2.3 (0.3–3.9)	
> median	18	1.3 (0.1–5.7)		154.0 (36.7–281.9)		1.6 (0.2–2.3)	

Clinico-pathological characteristics and tumor CK18-Asp396, total CK18 and CK18-Asp396/CK18 ratios. P-values were calculated with Kruskall-Wallis and Mann-Whitney tests and P ≤ 0.05 were considered significant, shown in bold. * Some values missing.

## Discussion

In the present study we found CK18-Asp396 and total CK18 levels in plasma from colorectal cancer patients to be related to patient and tumor characteristics, to change in relation to tumor resection, and to be a predictor for disease-free survival. The observation that the death of tumor cells generates detectable products in the circulation of cancer patients is interesting for diagnostics purposes and monitoring therapy that induces tumor cell death. Cytokeratins are abundantly present in epithelial cells and their expression is usually retained or even increased after oncogenic transformation [15]. CK18 is cleaved by caspase-3 during apoptosis, resulting in the release of the degraded CK18-Asp396 product, i.e., the M30 antigen, into the circulation. It has previously been shown that circulating CK18-Asp396 levels are elevated in patients with various epithelial cancer types and to be increased during chemotherapy [8,9,13].

The CK18-Asp396 and total CK18 levels in the plasma of colorectal cancer patients, before and after surgical resection of the tumor, correlated very well with each other, as expected, because the CK18 ELISA recognizes the soluble fragments of CK18 that are detected in the M30 ELISA, as well as other soluble non-caspase cleaved CK18 fragments. Both CK18-Asp396 and total CK18 plasma values increased shortly after surgical resection of the tumor, likely due to the surgical procedure. Because the apoptotic cells are randomly distributed throughout colorectal carcinomas [16], it is evident that products of apoptotic tumor cells do not all enter the circulation of these patients but are also released into the lumen and leave the body via stool, especially in tumors that have not invaded and spread to adjacent lymph nodes. Our results showed a correlation between CK18-Asp396 and total CK18 plasma levels and colorectal tumor stage, confirming that patients with advanced disease have higher CK18-Asp396 and total CK18 levels. The positive correlation of CK18-Asp396 plasma levels with tumor diameter further supports that the plasma levels are indeed elevated due to the presence of the tumor, with larger tumors responsible for more antigen "secretion". Because we found no correlation between tumor diameter or Dukes' stage with CK18-Asp396 level within the tumor, these parameters might be the cause of the inverse correlation between CK18-Asp396 plasma levels and CK18-Asp396 atumor levels. Taken together these results strongly indicate that CK18-Asp396 and total CK18 levels are reflected in the plasma of colorectal cancer patient due to the presence of the tumor.

CK18-Asp396 plasma levels were related to patient's outcome, independent from Dukes' stage, with patients with higher levels having worse disease-free survival. These observations are comparable to the reports about CK18-Asp396 levels in sera of patients with breast and lung cancer [8,9]. Patients with recurrent breast cancer also had highest CK18-Asp396 levels in their circulation and there was a correlation with the number of organs affected, suggesting increased CK18-Asp396 levels in the circulation associated with cancer progression [8], which concurs with our observation of a relation with Dukes' stage. Patients with lung cancer also had increased serum levels of CK18-Asp396, and patients with the lowest basal CK18-Asp396 levels showed the best survival [9]. Moreover, CK18-Asp396 and/or total CK18 levels were found to be increased due to chemotherapy in lung, prostate and breast cancer patients, showing the induction of tumor cell death [9-11,13]. In addition, the increase in total CK18 levels in breast cancer patients correlated with clinical response to therapy and survival. Thus, circulating CK18-Asp396 and total CK18 levels could potentially be used to monitor treatment efficacy in cancer patients. However, we did not find a relation between post-operative plasma CK18-Asp396 and/or total CK18 levels, in the plasmas obtained about 4.5 months after surgical intervention, and the post-operative adjuvant treatment the patients received, that started already within 1 month after surgery. Apparently, plasma CK18-Asp396 and/or total CK18 levels in colorectal cancer patients are intrinsically related to the tumor and less indicative for treatment response.

The ratio between plasma CK18-Asp396 and total CK18 levels reflects differences in apoptosis and necrosis, and might reflect tumor-related differences in those two cell death modes. Necrosis is believed to be a major process in hypoxic tumors as it does not need ATP to be executed, in contrast to apoptosis. Furthermore, hypoxia blocks apoptosis and contributes to treatment resistance [17,18]. Therefore, plasma CK18-Asp396/CK18 ratios can potentially be used to predict the response and determine which patients should be treated aggressively. In the present study, the CK18-Asp396/CK18 ratio tended to lower with increasing Dukes' stage, indicating that necrosis increases more than apoptosis during tumor progression, similar as reported for endometrial cancer [11]. High levels of necrosis in more advanced tumor stages fits with the idea that hypoxia forces the tumor to form new blood-vessels and to invade the muscularis mucosa to reach the circulation for oxygen supply, finally resulting in more advanced tumor stages [19,20]. Decreased plasma CK18-Asp396/CK18 ratios during tumor progression, furthermore, fit with the idea that there is a decrease in apoptotic sensitivity of tumor cells during colorectal tumor progression [16]. Interestingly, the patients CK18-Asp396 plasma levels and CK18-Asp396/CK18 ratios after tumor resection are also of prognostic relevance for the patient's disease-free survival. Both post-operative plasma CK18-Asp396 and CK18-Asp396/CK18 ratios were independent of the post-operative treatment the patients received. Thus, determination of plasma CK18-Asp396 and CK18-Asp396/CK18 ratios might also be a powerful independent tool to monitor patients after resection. In order to be conclusive, however, these interesting preliminary observations, due to the limited power of our study with only 49 colorectal cancer patients, merit further evaluation in larger patient groups.

## Conclusion

CK18-Asp396 and total CK18 levels in the circulation of colorectal cancer are prognostic for disease-free survival independent of disease stage, and might be helpful to select patient's treatment and in monitoring the patient after surgery, which should be confirmed in larger prospective studies.

## Competing interests

The authors declare that they have no competing interests.

## Authors' contributions

P.J.K, H.W.V. and C.B.H.W.L. gathered clinico-pathological data. P.J.K, C.B.H.W.L., D.W.H. and H.W.V. were involved in conception of the study, analysis of the data and interpretation of the results. P.J.K. and H.W.V. designed the study and wrote the manuscript. All authors approved the final manuscript.

## Pre-publication history

The pre-publication history for this paper can be accessed here:

http://www.biomedcentral.com/1471-2407/9/88/prepub

## Supplementary Material

Additional file 1 The data provided show that there is no relation between patient treatment and post-operative plasma CK18-Asp396, total CK18 and CK18-Asp396/CK18 ratios. Click here for file

Additional file 2 The data provided show that patients with high tumor CK18-Asp396, total CK18 levels or CK18-Asp396/CK18 ratios do not have a different disease-free than patients with low levels. Click here for file
