# Supplementary figures and images for: Circulating cell death products predict clinical outcome of colorectal cancer patients
Source: BMC Cancer. 2009 Mar 23;9:88. doi: 10.1186/1471-2407-9-88 (PMC2666761; doi:10.1186/1471-2407-9-88)

## Slide 1
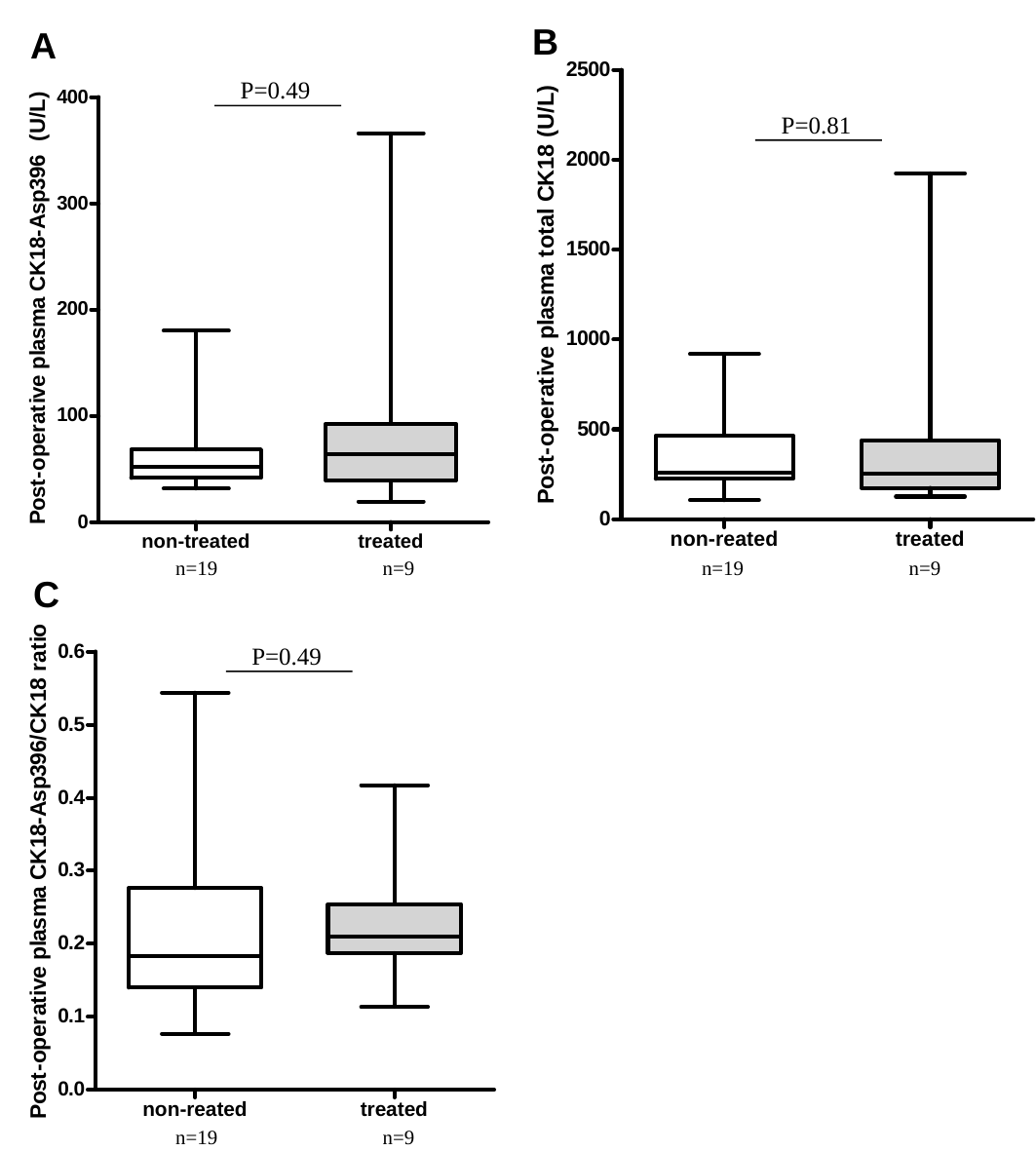

B
A
P=0.49
P=0.81
n=19
n=9
n=19
n=9
C
P=0.49
n=19
n=9

Supplement: Additional file 1 — The data provided show that there is no relation between patient treatment and post-operative plasma CK18-Asp396, total CK18 and CK18-Asp396/CK18 ratios. [file 1471-2407-9-88-S1.ppt]

## Slide 1
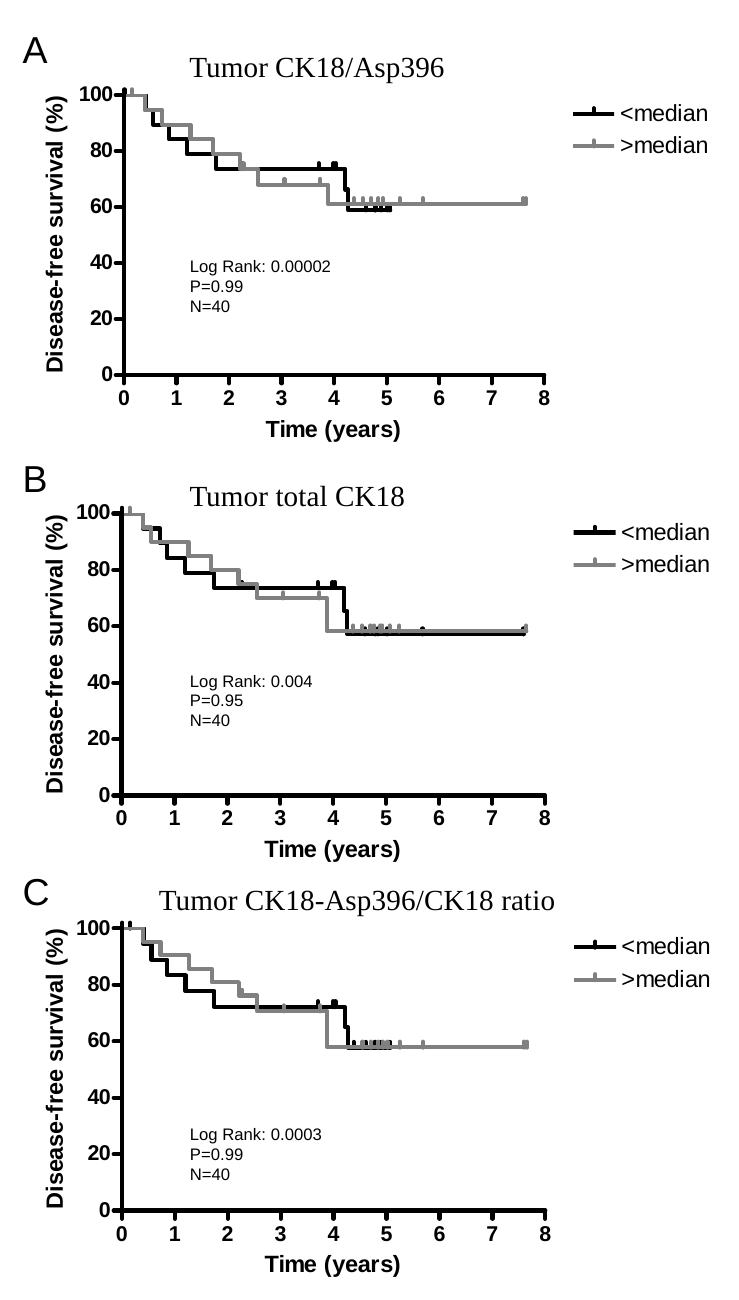

A
Tumor CK18/Asp396
Log Rank: 0.00002
P=0.99
N=40
B
Tumor total CK18
Log Rank: 0.004
P=0.95
N=40
C
Tumor CK18-Asp396/CK18 ratio
Log Rank: 0.0003
P=0.99
N=40

Supplement: Additional file 2 — The data provided show that patients with high tumor CK18-Asp396, total CK18 levels or CK18-Asp396/CK18 ratios do not have a different disease-free than patients with low levels. [file 1471-2407-9-88-S2.ppt]
